# Diseases of the Ear, Nose and Throat and Their Accessory Cavities

**Published:** 1899-06

**Authors:** 


DISEASES OF THE EAR, NOSE AND THROAT AND THEIR ACCESSORY CAVITIES. By Seth Scott Bishop, M.D., D.C.L., LL.D.; Professor of Diseases of the Nose, Throat, and Ear in the Illinois Medical College; Professor in the Chicago Post-Graduate Medical School and Hospital; Surgeon to the Post-Graduate Hospital; one of the Editors of the *Laryngoscope*, etc. Second Edition. Thoroughly revised and enlarged. Illustrated with ninety-four Chromo-Lithographs and two hundred and fifteen Half-tone and Photo-engravings. 6½ x 9½ inches. Pages xix-554. Extra Cloth, $4.00 net; Sheep or Half-Russia, $5.00 net. The F. A. Davis Co., Publishers, 1914-16 Cherry St., Philadelphia.

The second edition of this work is before us. Dr. Bishop is to be congratulated on its reception by the profession, which seems to have been universally favorable. In this second edition some new matter has been added, and several colored plates enhance its value to a marked degree. This book is especially commended to students, in that it treats the three organs, nose, throat and ear, together, which is the only correct arrangement. The work, of course, is not an exhaustive treatise, but the principles of treatment herein found can be safely followed. If there was any adverse criticism to be made it would be that the author speaks too much of "my method," and in some cases is not explanatory enough in his sentences. Some of the colored plates are beautiful, and the work throughout is profusely illustrated. This latter feature we regard as one of the best features of any text-book. R.

